# Cysteine residues are responsible for the sulfurous off-flavor formed in heated whey protein solutions

**DOI:** 10.1016/j.fochms.2022.100120

**Published:** 2022-07-12

**Authors:** Chengkang Li, Peter A. Paulsen, Halise Gül Akıllıoğlu, Søren B. Nielsen, Kasper Engholm-Keller, Marianne N. Lund

**Affiliations:** a Department of Food Science, Faculty of Science, University of Copenhagen, Rolighedsvej 26, 1958 Frederiksberg, Denmark; b Arla Foods Ingredients, Sønderupvej 26, 6920 Videbæk, Denmark; c Department of Biomedical Sciences, Faculty of Health and Medical Sciences, University of Copenhagen, Blegdamsvej 3, 2200 Copenhagen N, Denmark

**Keywords:** Volatile sulfur compound, Hydrogen sulfide, β-lactoglobulin, Beta-elimination, GC-FPD and LC-MS/MS

## Abstract

Odor-active volatile sulfur compounds are formed in heated food protein systems. In the present study, hydrogen sulfide (H_2_S) was found to be the most abundant sulfur volatile in whey protein solutions (whey protein isolate [WPI], a whey model system and single whey proteins) by gas chromatography-flame photometric detector (GC-FPD) analysis after heat treatments (60–90 °C for 10 min, 90 °C for 120 min and UHT-like treatment). H_2_S was detected in WPI after heating at 90 °C for 10 min, and was significantly increased at higher heat load (90 °C for 120 min and the UHT-like treatment). Site-specific LC-MS/MS-based proteomic analysis was conducted, monitoring desulfurization reactions in these protein systems to investigate the mechanism of H_2_S formation in heated WPI. Cysteine residues from beta-lactoglobulin were found to be responsible for the formation of H_2_S in heated WPI, presumably via beta-elimination.

## Highlights

• Formation of sulfur volatiles in heated whey protein (WP) solutions was investigated.
• H_2_S was found to be the primary volatile sulfur compound in heated WP solutions.
• Cys residues were found to be responsible for H_2_S formation in heated WP solutions.
• Beta-elimination of Cys residues was suggested to be the pathway of H_2_S formation.
• The total content of Cys residues (i.e. Cys and cystine) promoted H_2_S formation.

## 1 Introduction

Whey is the primary by-product of cheese manufacturing and the whey protein fraction is a versatile food ingredient with a high content of essential amino acids (Wijayanti, Bansal, & Deeth, 2014). Bovine whey is composed of about 50 % beta-lactoglobulin (β-LG), about 20 % alpha-lactalbumin (α-LA) and other low-abundance proteins (Fox et al., 2015, McSweeney and Fox, 2013, Walstra et al., 2005). Soluble whey protein aggregates are produced for many food applications by using thermal treatments to achieve desired functionalities such as heat stability for protein beverages (Ryan et al., 2012, Ryan and Foegeding, 2015). However, these thermal treatments also induce unpleasant (e.g. eggy, cabbage-like and sulfurous) flavors, which may be carried over or formed directly in the food product with a negative effect on consumer perception (Jansson et al., 2020, Wright et al., 2006).

The unpleasant flavors are mainly attributed to the formation of volatile sulfur compounds. Wright et al. (2006) suggested that dimethyl trisulfide (DMTS) was associated with the cabbage flavor, while eggy and sulfurous flavors are strongly correlated with the concentration of hydrogen sulfide (H_2_S) and carbon disulfide (CS_2_) (Jo et al., 2019, Vazquez-Landaverde et al., 2006). Sulfur-derived off-flavor was recognized by a sensory panel in reconstituted whey protein isolate (WPI, 3 % w/v) after 10 min of heating at temperatures of around 60–70 °C and above (Jansson et al., 2020). The significantly higher volatility of H_2_S (boiling point of −60 °C) compared to other sulfur volatiles potentially makes H_2_S an important aroma compound in heated whey protein systems.

The formation of H_2_S has been found to increase with higher concentrations of free thiol groups (-SH) adjusted by adding β-LG before heating the milk systems (Gaafar, 1987). β-LG is the major source of free thiol groups in WPI and milk and has therefore been suggested to be the primary H_2_S precursor (Hutton & Patton, 1952). Strecker degradation of free amino acid cysteine (Cys) in the presence of an α-dicarbonyl compound and thermal degradation of thiamine (vitamin B1) have been proposed to be pathways for H_2_S formation in heated milk by other authors (Al-Attabi et al., 2009, Hutton and Patton, 1952, Jo et al., 2018). However, a commercial WPI with negligible amounts of free amino acids and thiamine generated significant sulfurous off-flavors during heating (Jansson et al., 2020), indicating that free amino acids and thiamine were unlikely to be responsible for the formation of H_2_S in heat-treated WPI.

**Fig. 1:**
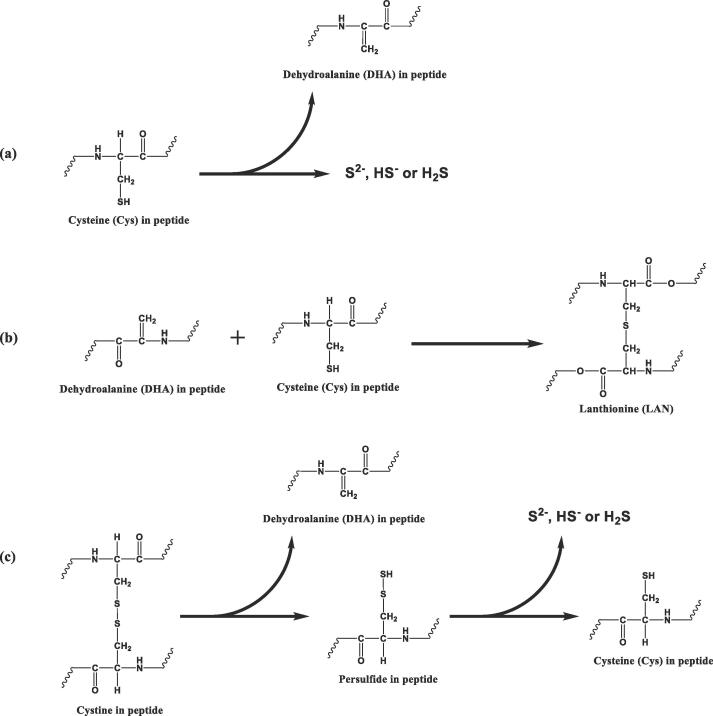
Cys β-Elimination of Protein-Bound Cys Residue (a), Michael Addition between Dehydroalanine (DHA) and Cys Residues (b), and β-Elimination of Protein-Bound Cystine and the Conversion of Persulfide into Cys Residue (c). Adapted with Modifications from (Nashef et al., 1977, Whitaker and Feeney, 1983).

As an alternative pathway to the Strecker degradation of Cys and thiamine degradation, Walstra et al. (2005) suggested a more likely pathway for heat-induced H_2_S formation in milk via β-elimination of protein-bound free Cys residues (referred to from now on as free Cys residues). In this pathway, low molecular weight thiol-containing compounds, such as free amino acids, are not required for the release of H_2_S. As a result of this Cys residue desulfurization reaction, a reactive electrophilic by-product (a dehydroalanine (DHA) residue) is also generated (Fig. 1**a**), which may undergo Michael addition with nucleophilic groups to form stable reaction products. For example, lanthionine (LAN) and lysinoalanine (LAL) are formed from reactions of the unsaturated double bond in DHA with the nucleophilic side chains of Cys and Lys, respectively (shown for LAN in Fig. 1**b**). These protein cross-links have been identified in various heat-treated food systems (Belitz et al., 2009, Fennema et al., 2017, Friedman, 1999, Lagrain et al., 2010).

The present study aimed to identify the origin and study the pathway of H_2_S formation in heated WPI. Thus, the generation of H_2_S was investigated in samples of WPI, a whey model system (mixture of α-LA and β-LG), α-LA alone, β-LG alone and sulfur-containing amino acids (methionine [Met] and Cys) after different heat treatments using a gas chromatography-flame photometric detector (GC-FPD). The role of β-elimination of Cys residues in H_2_S formation was evaluated by comparing the level of DHA residues and its downstream stable products, e.g. LAN and LAL, by site-specific LC-MS/MS-based proteomic analysis. In addition, the role of disulfide bonds on the formation of H_2_S in whey protein samples was also investigated, since Cys and cystine residues are known to rearrange due to thiol-disulfide exchange reactions, especially during heat treatments (Li, Engholm-Keller, & Lund, 2022).

## 2 Materials and methods

### 2.1 Chemicals and reagents

Powdered whey protein isolate (WPI) (Lacprodan® DI-9224, containing 89 % protein and < 0.2 % fat and lactose, based on dry matter) was obtained from Arla Foods Ingredients (Videbæk, Denmark). α-Lactalbumin (α-LA) from bovine milk (calcium-depleted, Lot # SLBT2518, 96 %, product # L6010), β-lactoglobulin (β-LG) from bovine milk variant A/B (Lot # SLBS6536, 99 %, product # L0130), l-Cys (97 %), l-Met (99 %), 4-(2-hydroxyethyl)-1-piperazineethanesulfonic acid (HEPES), 5,5′-dithiobis(2-nitrobenzoic acid) (DTNB), acetic acid, tris(2-carboxyethyl)phosphine (TCEP), iodoacetamide (IAA), sodium sulfide and TrypZean® bovine recombinant trypsin, sodium hydroxide, urea, sodium dihydrogen phosphate, di-sodium hydrogen phosphate, sodium acetate, endopeptidase Lys-C, and LiChroSolv LC-MS grade acetonitrile were obtained from Merck (Søborg, Denmark). *Staphylococcus aureus* endoproteinase-Glu-C was obtained from Worthington (Lakewood, NJ, USA). HiPerSolv LC/MS grade formic acid (99 %) was purchased from VWR (Søborg, Denmark). Double-deionized water was produced using a Milli-Q system (Millipore, Bedford, MA, USA). All reagents were of analytical grade.

### 2.2 Preparation of protein and amino acid samples

α-LA (2.45 mg/mL), β-LG (15.88 mg/mL), a whey model (2.45 mg/mL α-LA and 15.88 mg/mL β-LG), Cys (0.69 mg/mL), Met (0.54 mg/mL), sulfur amino acid mixture (0.69 mg/mL Cys and 0.54 mg/mL Met) and 3 % WPI (w/w) solutions were prepared in 10 mM phosphate buffer (pH 7.0) for GC-FPD analysis. All protein samples were also prepared in 55 mM HEPES buffer (pH 7.0) for proteomic and thiol analyses. Protein and amino acid concentrations in the prepared solutions were chosen to reflect their concentrations in a 3 % WPI (w/w) solution (Li, Nielsen, Engholm-Keller, & Lund, 2022).

### 2.3 Heat treatments for proteomic analysis

Samples (200 µL) in HEPES buffer were heated at 60, 70, 80, 90 °C for 10 min or 90 °C for 120 min in polymerase chain reaction (PCR) tubes (Molecular BioProducts Inc., San Diego, CA, USA) in a thermal cycler (Applied Biosystems 2720, version 2.09, Thermo Fischer Scientific Inc., Singapore) as previously described (Li, Engholm-Keller, & Lund, 2022). Heating at 90 °C for 10 min was representative for a typical industrial heat treatment used for producing functional protein aggregates, 90 °C for 120 min was considered as an extreme heat treatment. Another heat treatment at 160 °C for 160 s was chosen to mimic an ultra-high-temperature (UHT) treatment as preliminary data revealed formation of off-flavor levels corresponding to the levels produced in pilot-scale equipment for UHT treatment (data not shown), thus allowing more closely comparison to industry-relevant process conditions. In this UHT-like treatment, each 1 mL of sample solution in glass vials (item no. ML 33003 V, Mikrolab Aarhus A/S, Aarhus, Denmark) were sealed with silicone/polytetrafluoroethylene (PTFE) septa and crimp caps (item no. ML 33034L, Mikrolab Aarhus A/S, Aarhus, Denmark) and heated for 160 s in a pre-heated aluminum block (catalog no. 460–0008, VWR, Søborg, Denmark) at 160 °C. The samples were first cooled at room temperature for 1 min and then immersed in ice-water.

For proteomic analysis, protein samples were categorized into two groups (i.e. non-reduced and reduced groups) for investigation of different protein modifications. The pH of samples from the non-reduced group was adjusted to 6.5 with 50 mM sodium acetate (pH 6.0) after heat treatment. Then, free Cys residues were alkylated (via carbamidomethylation) using 15 mM IAA/4 M urea for 30 min in the dark (at 30 °C) before storing the samples at −20 °C. Samples from the reduced group were immediately frozen at −20 °C after heat treatment, while the reduction and alkylation processes were performed after the enzymatic hydrolysis as described in the “*2.5.2 LC-MS/MS*” section below.

### 2.4 Gas chromatography-flame photometric detector (GC-FPD) analysis

One milliliter of each untreated protein or amino acid sample in phosphate buffer was transferred to a sealed glass vial as described in the “*2.3 Heat Treatments for Proteomic Analysis*” section above. Samples were then heated following the abovementioned temperature/time combinations in the preheated aluminum block and stored at 10 °C before analysis on an Agilent GC7890B gas chromatography system equipped with a flame photometric detector (FPD Plus, Agilent Technologies Inc., Santa Clara, CA, USA). After five min incubation at 32 °C in the autosampler (G6501B GC Sampler 80, Agilent Technologies Inc., Santa Clara, CA, USA), 250 μL of headspace was injected through a splitless injection liner at 250 °C (single taper Ultra Inert (UI) with glass wool, 900 µL, Agilent Technologies Inc., Santa Clara, CA, USA). Analytes were separated on a polyethylene glycol (PEG) column (J&W DB-WAX Ultra Inert, 30 m × 0.25 mm, 0.25 μm, Agilent Technologies Inc., Santa Clara, CA, USA) with 1.2 mL/min hydrogen flow. The oven temperature was kept at 32 °C for six min, and increased to 230 °C at 50 °C/min for five min, followed by a five min final holding time. The FPD was operated in sulfur mode at 255 °C.

A H_2_S stock solution was prepared by dissolving 385 mg of sodium sulfide in 50 g of phosphate buffer (pH 8.5) (Vazquez-Landaverde et al., 2006). The stock solution was diluted into a series of different concentrations in phosphate buffer at pH 7.0 for the preparation of the standard curve. Data of the GC-FPD analysis was analyzed using MassHunter Quantitative Analysis software (ver. 10.1.49, Agilent Corp., Wilmington, DE, USA).

### 2.5 Quantification of total free thiols

The concentration of total free thiol groups in all protein samples was determined by the DTNB assay (Ellman, 1959). Protein samples were diluted appropriately and transferred (100 μL) to a 96-well-plate (Thermo Fisher Scientific Inc., Rochester, NY, USA), together with a series of freshly prepared Cys standard solutions (in 55 mM HEPES buffer [pH 7.0]) with concentration ranging between 0.1 and 1.0 mM. The working reagent was prepared by mixing 8 M urea in 10 mM phosphate buffer (pH 8.0) with freshly prepared 10 mM DTNB in 100 % ethanol at a 4:1 ratio. Working reagent (120 μL) was added to the wells containing the samples and standards and the plate was incubated at room temperature in the dark for 20 min. The absorbance at 412 nm was measured by a SpectraMax i3x Multi-Mode Microplate Reader (Molecular Devices, San Jose, CA, USA) at 30 °C. The concentration of total free thiols in the protein samples was calculated based on the calibration curve of Cys with the subtraction of the blank signal, which was the absorbance of the working reagent.

### 2.6 Proteomic analysis

#### 2.6.1 Specific enzymatic hydrolysis

β-LG samples from the non-reduced group were hydrolyzed by two times sequential addition of 2.5 % Glu-C (enzyme-to-protein ratio (w/w) 2.5:100) followed by 3 h and 15 h incubations, respectively. Other protein samples from the non-reduced group were hydrolyzed with 1 % Lys-C for 3 h, followed by hydrolysis using 5 % trypsin for 15 h. For all samples from the reduced group, pH and urea concentration were adjusted to 6.5 and 4 M, respectively, with an equal volume of 8 M urea/50 mM phosphate buffer (pH 6.0). A triple-protease sequential hydrolysis was then conducted with sequential addition of 1 % Lys-C (3 h), 5 % trypsin (15 h), and two times 2.5 % Glu-C (3 + 15 h). All enzymatic hydrolyses were conducted at pH 6.5 and 30 °C, and the urea concentrations were always diluted to 1 M with 50 mM sodium acetate (pH 6.5) after Lys-C hydrolysis.

#### 2.6.2 Liquid chromatography tandem mass spectrometry (LC-MS/MS) analysis

Samples from the reduced group were reduced using 13 mM TCEP and alkylated (via carbamidomethylation) using 30 mM IAA, with both incubations being conducted with shaking for 30 min at 25 °C in the dark. All digested samples were acidified with formic acid to a 5 % final concentration, transferred to a 96-well PCR plate (Axygen PCR-96-FS-C, VWR, Søborg, Denmark) with a seal mat (Axygen AM-96-PCR-RD, VWR, Søborg, Denmark), and stored at 10 °C before analysis. The LC-MS/MS analysis was conducted as previously described (Li, Engholm-Keller, & Lund, 2022) on a UHPLC Ultimate 3000 system equipped with a reversed-phase C18 column (Aeris 1.7 μm PEPTIDE XB-C18 100 Å, 100 × 2.1 mm, Phenomenex, Torrance, CA, USA) coupled with a Q Exactive hybrid quadrupole-Orbitrap mass spectrometer (MS) (Thermo Fisher Scientific Inc., Roskilde, Denmark).

#### 2.6.3 Modification database search on linear peptides

Peptide modifications of samples from the non-reduced group were identified by the Proteome Discoverer software (ver. 2.2.0.388, Thermo Fisher Scientific Inc., Roskilde, Denmark) with previously described settings (Li, Nielsen, Engholm-Keller, & Lund, 2022). Cys → DHA (DHA) (-34 Da), Cys → persulfide carbamidomethylation (persulfide) (+89 Da) and Cys carbamidomethylation (alkylation) (+57 Da) were chosen as variable modifications, while *N*-term → pyruvic acid (–33 Da) and C-term → amidation (-104 Da) were selected as variable modifications at any peptide *N*-terminus and C-terminus, respectively (Supporting Information, Figure S1). Database search results (.msf file) were validated in the Skyline software (ver. 20.1.0.76, University of Washington, Seattle, WA, USA) by manually checking retention time, isotopic correlation and fragment ion distribution for each modification (Li, Nielsen, Engholm-Keller, & Lund, 2022, Schilling et al., 2012). Databases containing β-LG (P02754) or α-LA (P00711) (downloaded from Uniprot [http://www.uniprot.org], accessed in September 2019) were selected for samples of β-LG and α-LA, respectively, while their combination was used for searches of the whey model and WPI.

#### 2.6.4 Modification database search for cross-linked peptides

Cross-linked peptides were identified and validated as previously described (Li, Engholm-Keller, & Lund, 2022) using pLink (ver. 2.3.9, Institute of Computing Technology, Chinese Academy of Sciences, Beijing, China) and Skyline (ver. 20.1.0.76, University of Washington, Seattle, WA, USA) software, respectively. Custom-defined linkers “LAN”, between two Cys residues (-34 Da); “LAL”, between Cys and Lys residues (-34 Da) and “histidinoalanine (HAL)” between Cys and His residues (-34 Da) were included in the pLink database search based on corresponding protein databases as mentioned above (Fan, Meng, Lu, Zhang, Yang, Chi, Sun, Dong, & He, 2015). Glu-C and/or trypsin were set as digestion enzyme(s) according to the proteolytic treatment and Cys carbamidomethylation (alkylation) (+57 Da) was selected as a variable modification. Relative quantification of cross-linked peptides was conducted for samples subjected to the reducing condition.

#### 2.6.5 Relative quantification and data analysis

Relative quantification of modifications and cross-links were performed for selected validated peptides in the Skyline software by integrating the area-under-curve (AUC) of the three first isotopologue ion peaks in the extracted ion chromatogram for the selected charge state that had the highest signal intensity. All peptide signals were normalized by using reference peptides as internal standards, ILDK, [95–98], (+1), from α-LA and either GLDIQK, [9–14], (+1), from β-LG under reducing condition, or KTKIPAVFKIDALNE, [75–89], (+3), from β-LG under non-reduced condition. Signals of these reference peptides remained constant (within the same order of magnitude), regardless of heat treatments and protein systems.

Assuming that H_2_S was released from free Cys residues, the extent of formation of H_2_S from Cys residue(s) in different heated protein samples was estimated by Equations (1) and (2), while the extent of disulfide cleavage during heat treatment was estimated by Equation (3).

When [free thiol]*_unheated_* > [free thiol]*_heated_*:

(1) $$The\;extent\;of\;formation\;of\;H_{2}S\;from\;Cys\;residues\;\left[\%\right]=\;\frac{{\left[H_{2}S\right]}_{\mathrm{heated}}}{{\left[free\;thiol\right]}_{\mathrm{unheated}}+\;{\left[H_{2}S\right]}_{\mathrm{heated}}}\times 100\%$$

where [H_2_S]*_heated_* = concentration of detected H_2_S in the heated sample and [free thiol]*_unheated_* = concentration of free thiol in the unheated sample. Due to the increased concentration of free thiol in certain protein solutions after heat treatments, the free thiol concentration of the unheated sample from Equation 1 was replaced by the free thiol concentration measured after the heat treatment ([free thiol]*_heated_*), while calculating the formation extent of H_2_S from Cys residue(s) to avoid over-estimation (Equation (2).

Thus, when [free thiol]*_heated_* > [free thiol]*_unheated_*:

(2) $$The\;extent\;of\;formation\;of\;H_{2}S\;from\;Cys\;residues\;\left[\%\right]=\;\frac{{\left[H_{2}S\right]}_{\mathrm{heated}}}{{\left[free\;thiol\right]}_{\mathrm{heated}}\;+\;{\left[H_{2}S\right]}_{\mathrm{heated}}}\times 100\%$$

(3) $$The\;extent\;of\;disulfide\;cleavage\;\left[\%\right]=\;\frac{{\left[free\;thiol\right]}_{\mathrm{heated}}\;-\;{\left[free\;thiol\right]}_{\mathrm{unheated}}\;}{2\;\times [theoretical\;cystine]}\times 100\%$$

where [theoretical cystine] = concentration of theoretical cystine in the sample before heating.

### 2.7 Quantification of lanthionine (LAN) after acidic hydrolysis

Quantification of LAN was performed by using a previously validated and published method (Akıllıoğlu & Lund, 2022). Briefly, aliquots (200 μL) of protein samples (1 mL aliquots were used for α-LA samples) were mixed with 6 M HCl (final concentration) and hydrolyzed in a microwave system (Biotage Initiator, Uppsala, Sweden) in 2 mL microwave reaction vials. The hydrolysate was evaporated to dryness using a centrifugal vacuum evaporator (Savant® SPD131DDA SpeedVac Concentrator, Thermo Fisher Scientific Inc., Roskilde, Denmark). Then the residue was dissolved in Milli-Q water and filtered through a 0.20 µm regenerated cellulose syringe filter (Phenomenex, Torrance, CA, USA). A stable isotopically labeled internal standard, lysine-d4, (Iris-Biotech, Marktredwitz, Germany) was added to each hydrolyzed sample, followed by mixing with an equal volume of acetonitrile. Five microliter of each sample was injected into the UHPLC-MS system, which was equipped with a Syncronis HILIC column (100 × 2.1 mm, 1.7 µm, Thermo Fischer Scientific Inc., Roskilde, Denmark), and analyzed following the chromatographic conditions and mass spectrometric details previously described by Akıllıoğlu and Lund (2022). The concentration of LAN in acid-hydrolyzed protein samples was calculated based on the linear calibration curve of external standards, i.e. 10–4,000 ng/mL LAN (Iris-Biotech, Marktredwitz, Germany), with normalization based on the signal of internal standard in each sample.

### 2.8 Statistical analysis

All heating (or control) experiments were performed as independent triplicates, unless otherwise mentioned, and results were shown as mean values ± standard deviation (SD). Limits of detection (LODs) were calculated according to the formula: LOD = 3.3 × (standard deviation of the standard curve/slope of the standard curve). Statistical analysis was performed by one-way ANOVA with a Tukey-Kramer HSD post-hoc test in JMP Pro 15 (SAS Institute Inc., Cary, North Carolina, USA). All MS.raw data in the present study was the same as in our previously published papers (Li et al., 2022, Li, Nielsen, Engholm-Keller, & Lund, 2022), where the extent of disulfide rearrangement and protein oxidation was investigated. The modification search results are available via MassIVE (https://massive.ucsd.edu) with identifier MSV000088811.

## 3 Results & discussion

### 3.1 β-LG and Cys are the key precursors of heat-induced H_2_S in WPI

Heat-induced formation of volatile sulfur compounds was investigated in WPI, whey proteins (α-LA and β-LG) and sulfurous amino acids (Cys and Met) by specific sulfur detection using GC-FPD. Among all tested protein and amino acid samples, H_2_S was the only detected volatile sulfur compound after all employed heat treatments. The H_2_S concentrations detected in the present study were above its reported sensory threshold (Table 1), which is ca. 0.3 μM in water (Rychlik, Schieberle, & Grosch, 1998), and were significantly (>3,000 times) higher than other volatile sulfur compounds (e.g. DMDS) detected in WPI after heat treatments (at up to 90 °C, 10 min) in a previous study (Jansson et al., 2020). These results demonstrate that H_2_S has a significant role in off-flavor formation in heated WPI solutions.

**Table 1:** GC-FPD Quantification of H_2_S (μM) in Heat-Treated Protein and Amino Acid Samples.#.

Sample	Heat Treatments				
	Unheated	80 °C (10 min)	90 °C (10 min)	90 °C (120 min)	UHT-like (160 °C, 160 s)
α-LA	<LOD	NT	<LOD	<LOD	<LOD
β-LG	<LOD	2.0 ± 0.1 ^*; a^	7.3 ± 0.2 ^**; c^	15.4 ± 0.7 ^***; c^	22.2 ± 1.8 ^****; a^
Whey model	<LOD	2.9 ± 0.5 ^*; b^	9.5 ± 0.6 ^**; d^	18.7 ± 1.0 ^***; d^	30.1 ± 3.0 ^****; a,b^
WPI	<LOD	<LOD	4.0 ± 0.1 ^*; b^	23.3 ± 0.4 ^**; e^	38.1 ± 8.6 ^***; a,b^
Concentrated α-LA	<LOD	NT	1.5 ± 0.2 ^*; a^	4.0 ± 0.3 ^*; a^	51.6 ± 17.9 ^**; c^
Cys	<LOD	NT	3.8 ± 0.3^b^	10.7	44.8
Met	<LOD	NT	<LOD	<LOD	<LOD
Mixed amino acid	<LOD	NT	4.3 ± 0.6 ^*; b^	11.6 ± 0.8 ^**; b^	NT

# Protein concentrations of samples: α-LA: 2.45 mg/mL (0.17 mM); β-LG: 15.88 mg/mL (0.87 mM); whey model: 2.45 mg/mL (0.17 mM) α-LA + 15.88 mg/mL (0.87 mM) β-LG; WPI: 30 mg/mL and conc. α-LA: 16 mg/mL (1.11 mM). Amino acid concentrations of samples: Cys: 0.69 mg/mL (5.7 mM); Met: 0.54 mg/mL (3.6 mM) and mixed amino acid: 0.69 mg/mL (5.7 mM) Cys + 0.54 mg/mL (3.6 mM) Met. Values in the same row followed by different number of asterisks (*) are significantly different between treatments, and values in the same column followed by different letters are significantly different between samples (one-way ANOVA with a Tukey-Kramer HSD post-hoc test, p < 0.05), tests were conducted with at least two independent replicates, while only one measurement was performed for the values without standard deviation. NT, not tested; LOD = 1.3 µM.

In WPI, H_2_S was detected after heat treatment at 90 °C for 10 min at a concentration of 4.0 ± 0.1 μM, which significantly increased to 23.3 ± 0.4 and 38.1 ± 8.6 μM after heating at 90 °C for 120 min and the UHT-like treatment, respectively. The detected H_2_S concentrations in the present study were also much higher than the values found in most heated milk products as summarized by Al-Attabi et al. (2009). The gas phase of the samples in the present study was injected directly into the GC-FPD system, which minimized the loss of volatile compounds that could potentially occur during the additional extraction step used in previous studies for H_2_S quantification. The limited amount (below the limit of detection of the method [LOD = 1.3 µM]) of H_2_S in 80 °C (10 min) heated WPI samples observed in the present study may partially explain the significantly different levels of aromas that were perceived by smell between 80 and 90 °C (10 min) heated WPI in the study of Jansson et al. (2020). However, H_2_S was detected in both heated pure β-LG and the whey model system (α-LA + β-LG) with heating at ≥ 80 °C (10 min). Interestingly, more H_2_S was generated in the heated whey model (9.5 ± 0.6 µM) and β-LG (7.3 ± 0.2 µM) solutions at 90 °C for 10 min than in WPI (4.0 ± 0.1 µM), whereas a higher H_2_S content was found in WPI at higher heat loads (90 °C for 120 min and the UHT-like treatment) compared to the whey model and β-LG (Table 1). Regardless of the heat treatment, H_2_S was absent in all heated samples of pure α-LA at a concentration similar to that of the (3 %) WPI sample (2.45 mg/mL) indicating that H_2_S was mainly formed from β-LG in the whey model and WPI. Increasing H_2_S concentrations were observed in Cys-containing amino acid solutions, i.e. Cys and the sulfur amino acid mixture (Cys + Met), with increasing heat load, whereas no H_2_S was detected in any of the heated solutions containing only Met (Table 1). Altogether, these results showed that β-LG and Cys were the key precursors of heat-induced H_2_S in WPI, which was in line with the findings of Gaafar (1987), where H_2_S formation was positively correlated with the quantity of free Cys residues and the heat load. Because free Cys amino acids were not detected in WPI (Jansson et al., 2020), β-LG, being the primary source of free Cys residue (with free thiol groups) in WPI, is therefore suggested to be responsible for the detected H_2_S in heated WPI.

In addition, according to the proteomic analysis, signals of protein-bound DHA were identified site-specifically at all Cys residues in β-LG, with significant increases after severe heat treatments (i.e. 90 °C for 120 min and the UHT-like treatment) (Table 2). This increasing DHA signal was also detected at Cys66 and Cys160 on β-LG in both mixed protein systems (i.e. the whey model and WPI). Hence, H_2_S released from the heated whey protein solutions was suggested to be generated from (protein-bound) free Cys residues (with free thiol groups), presumably via Cys β-elimination (Fig. 1**a**) (Gaafar, 1987, Jo et al., 2018, Walstra et al., 2005). The frequently-used unspecific method for DHA determination that relies on conversion of DHA into pyruvic acid was not used in the present study, because DHA could also be formed at other amino acid side chains, e.g. serine (Ser), via β-elimination, without generating H_2_S (Rombouts, Lagrain, Brijs, & Delcour, 2011).

**Table 2:** LC-MS/MS Relative Quantification of Peptides-Bound Dehydroalanine (DHA) and Persulfide in Heat-Treated Protein Samples.*.

Protein	Cys residue	Modification	Sample	Heat Treatment				
				Unheated	80 °C, 10 min	90 °C, 10 min	90 °C, 120 min	UHT-like (160 °C, 160 s)
α-LA	Cys6	Persulfide	α-LA	4.5 ± 0.4 % ^a^	5.7 ± 0.9 % ^a^	6.5 ± 0.5 % ^a,b^	19.3 ± 2.2 % ^b,c^	100.0 ± 11.7 % ^f^
			whey model	14.5 ± 3.8 % ^a,b,c^	7.0 ± 1.1 % ^a,b^	13.0 ± 1.5 % ^a,b^	34.7 ± 4.2 % ^d^	75.3 ± 13.0 % ^e^
			WPI	ND	3.7 ± 0.8 % ^a^	9.9 ± 1.9 % ^a,b^	26.9 ± 3.3 % ^c,d^	81.7 ± 8.8 % ^e^
	Cys28	DHA	α-LA	ND	ND	ND	9.1 ± 1.6 % ^a^	100.0 ± 13.3 % ^b^
			whey model	ND	ND	ND	ND	ND
			WPI	ND	ND	ND	ND	ND
	Cys61	DHA	α-LA	0.2 ± 0.03 % ^a^	1.4 ± 0.4 % ^a^	3.2. ± 0.5 % ^a^	21.1 ± 4.0 % ^b^	100.0 ± 20.7 % ^d^
			whey model	0.1 ± 0.03 % ^a^	1.2 ± 0.2 % ^a^	2.6 ± 0.4 % ^a^	11.1 ± 1.7 % ^a,b^	56.0 ± 11.0 % ^c^
			WPI	ND	1.7 ± 0.3 % ^a^	3.4 ± 0.6 % ^a^	12.1 ± 2.0 % ^a,b^	49.3 ± 10.0 % ^c^
		Persulfide	α-LA	0.7 ± 0.1 % ^a^	1.8 ± 0.2 % ^a^	2.7 ± 0.2 % ^a^	16.4 ± 1.6 % ^b^	100.0 ± 11.8 % ^e^
			whey model	0.9 ± 0.5 % ^a^	6.4 ± 0.7 % ^a,b^	7.0 ± 1.1 % ^a,b^	10.4 ± 1.1 % ^a,b^	74.4 ± 11.9 % ^d^
			WPI	ND	ND	1.6 ± 0.4 % ^a^	6.8 ± 0.7 % ^a,b^	50.6 ± 9.1 % ^c^
	Cys73/Cys77	DHA and Alkylation	α-LA	0.0 ± 0.0 % ^a^	3.8 ± 0.5 % ^a,b^	8.3 ± 0.7 % ^a,b,c^	13.1 ± 1.7 % ^c^	62.9 ± 6.5 % ^e^
			whey model	ND	0.6 ± 0.1 % ^a,b^	2.7 ± 0.3 % ^a,b^	9.3 ± 1.1 % ^b,c^	100.0 ± 9.6 % ^f^
			WPI	ND	ND	ND	ND	52.2 ± 10.9 % ^d^
		2 × DHA	α-LA	0.1 ± 0.0 % ^a^	0.2 ± 0.1 % ^a^	0.5 ± 0.1 % ^a^	10.6 ± 2.5 % ^a,b^	100.0 ± 26.8 % ^d^
			whey model	ND	ND	ND	1.11 ± 0.56 % ^a^	48.1 ± 12.7 % ^c^
			WPI	ND	ND	ND	ND	25.5 ± 12.4 % ^b^
	Cys91	DHA	α-LA	0.2 ± 0.0 % ^a^	1.2 ± 0.1 % ^a^	3.0 ± 0.2 % ^a,b^	11.5 ± 0.8 % ^c^	100.0 ± 9.4 % ^f^
			whey model	ND	0.2 ± 0.4 % ^a^	3.4 ± 0.5 % ^a,b^	11.9 ± 1.0 % ^c^	53.8 ± 4.0 % ^d^
			WPI	ND	0.4 ± 0.6 % ^a^	3.3 ± 0.2 % ^a,b^	7.9 ± 2.5 % ^b,c^	77.5 ± 6.1 % ^e^
	Cys111	DHA	α-LA	0.4 ± 0.2 % ^a^	1.3 ± 0.1 % ^a,b^	2.2 ± 0.2 % ^a,b^	10.5 ± 0.7 % ^b^	100.0 ± 7.7 % ^d^
			whey model	ND	0.7 ± 0.0 % ^a^	1.6 ± 0.1 % ^a,b^	6.9 ± 0.5 % ^a,b^	74.5 ± 10.8 % ^c^
			WPI	ND	1.0 ± 0.7 % ^a,b^	1.5 ± 0.1 % ^a,b^	5.5 ± 0.6 % ^a,b^	70.3 ± 8.7 % ^c^
		Persulfide	α-LA	1.3 ± 0.5 % ^a^	2.40 ± 0.49 % ^a^	4.2 ± 0.4 % ^a,b^	17.3 ± 1.4 % ^b^	100.0 ± 8.7 % ^d^
			whey model	ND	ND	ND	ND	73.3 ± 11.2 % ^c^
			WPI	ND	ND	ND	ND	62.7 ± 15.9 % ^c^
	Cys120	DHA	α-LA	1.2 ± 0.2 % ^a^	1.6 ± 0.2 % ^a^	2.3 ± 0.3 % ^a^	9.1 ± 1.2 % ^a,b^	66.6 ± 10.4 % ^c^
			whey model	2.5 ± 0.4 % ^a^	2.9 ± 0.3 % ^a,b^	3.7 ± 0.4 % ^a,b^	12.2 ± 1.9 % ^b^	61.1 ± 8.3 % ^c^
			WPI	0.6 ± 0.1 % ^a^	1.1 ± 0.2 % ^a^	2.1 ± 0.2 % ^a^	9.4 ± 1.2 % ^a,b^	100.0 ± 13.9 % ^d^
		Persulfide	α-LA	6.8 ± 1.3 % ^a^	8.1 ± 1.6 % ^a^	8.5 ± 1.6 % ^a^	14.7 ± 2.7 % ^a^	71.2 ± 14.6 % ^b,c^
			whey model	36.1 ± 12.1 % ^b^	14.1 ± 2.8 % ^a^	11.4 ± 2.1 % ^a^	15.1 ± 3.1 % ^a^	100.0 ± 25.3 % ^d^
			WPI	9.9 ± 2.3 % ^a^	4.8 ± 0.9 % ^a^	4.7 ± 1.0 % ^a^	10.6 ± 1.9 % ^a^	87.3 ± 19.5 % ^c,d^
β-LG	Cys66	DHA	β-LG (Glu-C)	ND	10.0 ± 1.8 % ^a^	14.9 ± 3.2 % ^a^	46.3 ± 7.9 % ^b^	100.0 ± 23.0 % ^c^
			whey model	ND	0.3 ± 0.1 % ^a^	1.8 ± 0.2 % ^a^	13.9 ± 2.4 % ^b^	100.0 ± 15.9 % ^d^
			WPI	ND	0.3 ± 0.2 % ^a^	2.4 ± 0.3 % ^a^	14.3 ± 3.4 % ^b^	68.7 ± 8.3 % ^c^
		Persulfide	β-LG (Glu-C)	15.2 ± 2.9 % ^a^	23.2 ± 4.5 % ^a,b^	29.9 ± 5.6 % ^a,b^	42.0 ± 8.1 % ^b^	100.0 ± 26.0 % ^c^
			whey model	ND	ND	ND	ND	ND
			WPI	ND	ND	ND	ND	ND
	Cys106	DHA	β-LG (Glu-C)	ND	ND	ND	ND	100.0 ± 39.8 %
			whey model	ND	ND	ND	ND	ND
			WPI	ND	ND	ND	ND	ND
		Persulfide	β-LG (Glu-C)	0.1 ± 0.0 % ^a^	0.5 ± 0.1 % ^a^	1.2 ± 0.2 % ^a^	2.1 ± 0.6 % ^a^	100.0 ± 19.1 % ^b^
			whey model	ND	ND	ND	ND	ND
			WPI	ND	ND	ND	ND	ND
	Cys119 & Cys121	DHA and Alkylation	β-LG (Glu-C)	ND	ND	ND	ND	100.0 ± 24.8 % ^a^
			whey model	ND	ND	ND	ND	ND
			WPI	ND	ND	ND	ND	ND
	Cys160	DHA	β-LG (Glu-C)	3.4 ± 0.9 % ^a^	3.9 ± 1.0 % ^a^	6.1 ± 1.6 % ^a^	25.2 ± 6.4 % ^a^	100.0 ± 35.7 % ^b^
			whey model	0.4 ± 0.1 % ^a^	2.2 ± 0.3 % ^a^	4.5 ± 0.5 % ^a^	23.9 ± 1.9 % ^b^	80.6 ± 11.1 % ^c^
			WPI	0.4 ± 0.0 % ^a^	2.2 ± 0.2 % ^a^	4.3 ± 0.4 % ^a^	21.6 ± 1.9 % ^b^	100.0 ± 10.4 % ^d^
		Persulfide	β-LG (Glu-C)	8.9 ± 2.1 % ^a^	8.3 ± 2.0 % ^a^	8.2 ± 1.8 % ^a^	9.8 ± 2.5 % ^a^	100.0 ± 30.0 % ^b^
			whey model	2.9 ± 0.5 % ^a^	8.5 ± 1.8 % ^a,b^	11.2 ± 1.7 % ^a,b^	18.5 ± 1.5 % ^b^	96.1 ± 14.8 % ^c^
			WPI	ND	3.1 ± 0.4 % ^a^	4.2 ± 0.4 % ^a,b^	14.3 ± 1.2 % ^a,b^	100.0 ± 11.3 % ^c^

*The results of DHA and persulfide residues are presented as percentage values (the normalized peak area of the DHA- [or persulfide-] containing peptide for a particular treatment divided by the highest normalized peak area of the same residue peptide among all treatments and samples that followed the same digestion process) with their respective standard deviations (SDs) from the non-reduced sample group. Data from β-LG was Glu-C digested, as indicated as “β-LG (Glu-C)”, while all other samples were digested by Lys-C and trypsin. Values from β-LG (Glu-C) in the same modification (row) followed by different letters are significantly different between treatments. Other values from the same modification at the same Cys residue(s) followed by different letters are significantly different between treatments and systems. (One-way ANOVA with a Tukey-Kramer HSD post-hoc test, p < 0.05, n = 3). ND, not detected; Alkylation, Cys carbamidomethylation (+57 Da).

### 3.2 The total content of Cys and cystine affects H_2_S formation

To examine the effect of protein concentration on H_2_S formation, the GC-FPD based H_2_S analysis was also carried out on a concentrated (16 mg/mL) α-LA sample. After heating at 90 °C for 10 min, a limited concentration (1.5 ± 0.2 μM) of H_2_S was detected, yet it was above the reported sensory detection threshold (ca. 0.3 μM) and perceived by smell (Rychlik et al., 1998). The level of H_2_S was surprisingly high in the UHT-like treated concentrated α-LA (52 ± 18 μM), and was significantly higher than the H_2_S formed in the UHT-like-treated β-LG (30 ± 3 μM) with a comparable protein concentration (w/v) (Table 1). It was therefore speculated that the total content of Cys residues (including both free Cys residues and Cys present as disulfides) within the protein system was important for the formation of H_2_S; free Cys residues were absent in the unheated α-LA, but α-LA contained higher quantity of total Cys residues as compared to (the free Cys-containing) β-LG at the same protein concentration (w/v).

### 3.3 Beta-Elimination of cystine as a pathway of free Cys residue release

The total content of free Cys residue(s) in the protein samples was determined as free thiol concentration (Table 3). The increased thiol concentration observed in α-LA after heating at 90 °C for 120 min and the UHT-like condition suggested that disulfide bonds were cleaved in these samples, which was in agreement with a previous study (Nielsen, Lund, Davies, Nielsen, & Nielsen, 2018). These results also supported the findings from our previous study, in which disulfide rearrangement was observed in α-LA and pre-alkylated β-LG protein solutions heated at 90 °C for 120 min, but not after heating for 10 min (up to 90 °C) (Li, Engholm-Keller, & Lund, 2022). In UHT-like-treated β-LG-containing protein samples, a significant increase of free Cys residue (as free thiol) concentration was also observed (Table 3).

**Table 3:** Quantification of Thiol Concentrations (mM) in Heat-Treated Protein Samples.#.

Samples	Heat Treatments					
	Unheated	70 °C, 10 min	80 °C (10 min)	90 °C (10 min)	90 °C (120 min)	UHT-like (160 °C, 160 s)
α-LA	<LOD	NT	<LOD	0.00 ± 0.01 *	0.01 ± 0.01 *	0.02 ± 0.01 ^**^
Conc. a-LA	0.01 ± 0.01 *	NT	0.01 ± 0.01 *	0.01 ± 0.01 *	0.05 ± 0.01 ^**^	0.12 ± 0.01 ^***^
β-LG	0.78 ± 0.06 ^**^	0.77 ± 0.06 ^**^	0.75 ± 0.06 ^**^	0.73 ± 0.06 ^**; a,b^	0.48 ± 0.06 ^*; a^	1.20 ± 0.14 ^***; b^
Whey model	0.77 ± 0.05 ^**^	0.77 ± 0.05 ^**^	0.74 ± 0.05 ^**^	0.70 ± 0.05 ^**; a^	0.45 ± 0.05 ^*; a^	1.07 ± 0.12 ^***; a^
WPI	0.77 ± 0.06 ^**^	0.76 ± 0.06 ^**^	0.77 ± 0.06 ^**^	0.78 ± 0.06 ^**; b^	0.60 ± 0.06 ^*; b^	1.09 ± 0.07 ^***; a,b^

# Protein concentrations of samples: α-LA: 2.45 mg/mL (0.17 mM); conc. α-LA: 16 mg/mL (1.11 mM); β-LG: 15.88 mg/mL (0.87 mM); whey model: 2.45 mg/mL (0.17 mM) α-LA + 15.88 mg/mL (0.87 mM) β-LG, and WPI: 30 mg/mL. Values in the same row followed by different number of asterisks (*) are significantly different between treatments, and in β-LG-containing samples, values in the same column followed by different letters are significantly different between different β-LG-containing samples (one-way ANOVA with a Tukey-Kramer HSD post-hoc test, p < 0.05, n = 3). NT, not tested. LOD = 0.003 mM.

The release of free Cys residues from disulfide bonds in α-LA, which contained no free Cys residue in the unheated structure, could be due to β-elimination occurring at disulfide bonds, as previously studied in other proteins under thermal treatment (Lagrain et al., 2010, Rombouts et al., 2010, Volkin and Klibanov, 1987). The formed (protein-bound) persulfide from cystine β-elimination can then be converted into free Cys residue with the potential release of H_2_S (Fig. 1**c**) (Friedman, 1999). According to the sensitive LC-MS/MS-based proteomic analysis, the two main (intermediate) reaction products of cystine β-elimination, DHA and persulfide (Nashef et al., 1977), were site-specifically detected in peptides originating from both α-LA and β-LG in all heated protein samples under the non-reduced conditions (Table 2). More specifically, DHA was identified in both heated single whey proteins (i.e. α-LA and β-LG) at almost all Cys residues with increasing levels as the heat load increased, being significant at the intense conditions (i.e. 90 °C, 120 min and the UHT-like treatment). Persulfide was found at many Cys residues and shared a similar increasing pattern with the DHA signal (Table 2). Most of the DHA signals found in the single whey proteins were also detected in the mixed protein systems, i.e. the whey model and WPI, except on two relatively long Cys-containing peptides, i.e. [17–58] from α-LA and [102–124] from β-LG. It was hypothesized that these long peptides, having multiple reactive amino acids, may have a high possibility to undergo different modifications (e.g. oxidation) simultaneously under elevated temperatures, leading to a lower probability of having only Cys modified to DHA, especially in α-LA (Li, Nielsen, Engholm-Keller, & Lund, 2022). In addition, the tryptic β-LG tri-Cys peptide [102–124] from mixed protein systems under the non-reduced condition was likely to be involved in disulfide-linked peptides, resulting in the absence of signal of the targeted linear DHA-modified peptide in the present proteomic analysis. The matrix effect in these two complex samples (i.e. the whey model and WPI), as compared to the single protein solutions, potentially also suppressed the detection of DHA and persulfide on these two long peptides (i.e. [17–58] from α-LA and [102–124] from β-LG) in the LC-MS/MS analysis (Jessome & Volmer, 2006), especially when these reactive intermediates were in low quantity.

The Cys residues that were detected in modified forms as either DHA or persulfide covered all Cys residues from α-LA and β-LG, while some were even present in the unheated controls (Table 2). This indicated that non-native free Cys residues were released (presumably via cystine β-elimination/thiol-disulfide exchange) during the applied heat treatments and/or to some extent during the protein production/purification, in which heat/alkaline treatment(s) were possibly involved (Nashef et al., 1977, Whitaker and Feeney, 1983).

### 3.4 Formation of DHA-Derived protein Cross-Links

Potential DHA-derived nucleophilic addition products resulting in peptide cross-links (e.g. LAN and LAL) were targeted and quantified in different protein systems via proteomic analysis under reducing conditions (Supporting Information, Table S1). Unlike DHA, signals of LAN and LAL were rarely present after heat treatments conducted for 10 min, but mainly appeared after 90 °C heating for 120 min and the UHT-like treatment. All Cys residues originating from α-LA (at the low concentration) were shown to participate in LAN formation, with nine different linkages identified, although the H_2_S signal was absent in heated α-LA at the low concentration. Thus, LAN detected in heated α-LA was suggested to form at the protein-bound DHA that was generated via β-elimination at disulfide-linked cystine residues (Fig. 1**b and c**). In β-LG-containing samples, where H_2_S was detected, only one LAN was detected linking Cys66 and Cys106 (Supporting Information, Table S1). Presumably, the use of the triple-protease-hydrolysis procedure in reduced β-LG samples might have increased the sample complexity, making it challenging to identify these low-abundant cross-links from β-LG on their fully-cleaved peptides. With Lys being both the cleavage site of trypsin and the cross-linking site of LAL, LAL-linked peptides were expected to be found in relatively long mis-cleaved peptides. Thus, only one LAL was identified in heated α-LA (Cys91-Lys98), while a histidinoalanine (HAL) cross-link, as a Michael addition product of DHA and a His residue, was also detected in the intensely heated α-LA, linking Cys91 and His107. The detection of HAL further supported the occurrence of β-elimination at cystine (and/or Cys) residues in the heated protein systems.

The absolute quantities of LAN were also determined in acid-hydrolyzed protein samples by LC-MS with standards in known concentrations (Table 4). The content of LAN was found to increase with increasing heat load in all protein samples, which was in agreement with the findings from the proteomic analysis. LAN concentrations detected in the whey model and WPI were significantly higher than in β-LG, and much higher than in α-LA, after intense heat treatments, implying different extents of β-elimination in different protein systems.

**Table 4:** LC-MS/MS Quantification of LAN (μM) in Heat-Treated Protein Samples after Acid Hydrolysis.#.

Sample	Heat treatment				
	Unheated	80 °C (10 min)	90 °C (10 min)	90 °C (120 min)	UHT-like (160 °C, 160 s)
α-LA	<LOD	<LOD	<LOD	1.0	1.5
β-LG	<LOD	<LOD	<LOD	3.9 ± 0.5 ^**; a^	4.2 ± 0.9 ^**; a^
Whey model	<LOD	1.6 ± 0.6 ^*; a^	2.0 ± 0.4 ^*; a^	11.8 ± 1.7 ^**; b^	14.4 ± 2.1 ^**; b^
WPI	<LOD	1.3 ± 0.1 ^*; a^	2.6 ± 0.6 ^*; a^	11.5 ± 1.3 ^**; b^	21.4 ± 5.0 ^***; b^

# Protein concentrations of each sample: α-LA: 2.45 mg/mL (0.17 mM); β-LG: 15.88 mg/mL (0.87 mM); whey model: 2.45 mg/mL (0.17 mM) α-LA + 15.88 mg/mL (0.87 mM) β-LG, and WPI: 30 mg/mL. Values in the same row followed by different number of asterisks (*) are significantly different between treatments, and values in the same column followed by different letters are significantly different between samples (one-way ANOVA with a Tukey-Kramer HSD post-hoc test, p < 0.05), values of α-LA were obtained from the mixture of five independent heat treatments. LOD = 0.4 μM.

The formation of H_2_S and LAN in heated protein solutions followed the same trend (Supporting Information, Figure S2). The formation of LAN was likely to be an important reaction for the DHA that was formed via Cys β-elimination accompanied by the formation of H_2_S. However, DHA could also be formed on other amino acid residues than Cys (e.g. Ser) without generating H_2_S. It was found that the concentration of the released free Cys residues (mM level) (Table 3) was significantly (∼1,000 times) higher than the measured LAN concentration (μM level) (Table 4) after heat treatments. It was therefore suggested that other possible pathways existed for disulfide breakage and release of free Cys residues (without generating DHA) in heated whey proteins, and/or that other reaction(s) of the newly-formed DHA took place (e.g. formation of LAL). For example, oxidative breakage of the amide bond (Supporting Information, Figure S1) was reported to occur at protein-bound DHA (Hellwig, 2019, Hellwig et al., 2015, Herbert et al., 2003, Tian et al., 2018), leading to protein fragmentation as observed previously by SDS-PAGE (Li, Nielsen, Engholm-Keller, & Lund, 2022). The resulting amide and pyruvoyl groups of this proposed reaction were both specifically identified at Cys residues (i.e. Cys6, Cys28, Cys73, Cys77, Cys91 and Cys120 on α-LA, and Cys66, Cys106, Cys119 and Cys121 on β-LG) in our current study with signals significantly increased in protein systems after the UHT-like treatment (data not shown).

### 3.5 The release and consumption of free Cys residues

β-Elimination has previously been proposed to take place at both Cys and cystine residues under heat and/or alkaline conditions (Nashef et al., 1977, Volkin and Klibanov, 1987, Whitaker and Feeney, 1983). Because Cys and cystine residues are interconvertible via disulfide rearrangement, it is not possible to state whether the detected DHA signal on Cys residues in our study originated from direct β-elimination of Cys residues or (re-arranged) cystine residues. However, by comparing the release of free Cys residues, as a result of cystine breakage, and the generated H_2_S content after heat treatments, the extent of H_2_S formation from Cys residues (likely via β-elimination) (**Eq. (1)** and (**2)**) and of disulfide-linked cystine breakage (**Eq. (3)**) can therefore be evaluated in different heated protein systems. In this case, the free Cys content can be monitored via the concentration of free thiol (Table 3). The estimated extent of disulfide cleavage was found to be comparable in the heated α-LA, regardless of protein concentration (Table 5**a**), although H_2_S was only detected in intensely heated (≥90 °C) concentrated α-LA (Table 1). It was therefore assumed that the released free Cys residue content in the diluted α-LA sample with a concentration similar to the 3 % WPI (2.45 mg/mL) was not high enough to form detectable amounts of H_2_S, and/or that other Cys reactions were more favorable under the applied heat treatments. Such other Cys reactions, e.g. thiol-disulfide exchange, Cys oxidation, and LAN formation, were likely competing with H_2_S formation for the limited free Cys residues within the system (Li et al., 2022, Li, Nielsen, Engholm-Keller, & Lund, 2022). This was supported by the fact that the much higher initial free Cys concentration in the pure Cys amino acid solution did not always generate higher levels of H_2_S after heat treatment compared to WPI that was heated under the same conditions (Table 1). In addition, the significant loss of free thiol in all β-LG containing samples heated at 90 °C for 120 min was clearly higher than the concentration of formed H_2_S after the same treatment (Table 1, Table 3), which supported the suggestion of other Cys reactions occurring in parallel. As calculated from **Equations** (**1)** and (**2)**, the formation of H_2_S only accounted for a small (<5%) fraction of all Cys reactions, and the extent of this reaction was estimated to be higher in the more complex samples that contained β-LG (WPI > whey model > β-LG) under severe heat treatments (90 °C, 120 min and the UHT-like treatment) (Table 5**b**). However, the significantly higher calculated extent of H_2_S formation from free Cys residues in the intensely heated concentrated α-LA sample, compared to other whey protein solutions following the same heat treatments (Table 5**b**), was likely to suggest other pathways of H_2_S formation, besides Cys β-elimination. One possible explanation for this H_2_S release could be persulfide degradation (Fig. 1**c**), but this requires further investigation.

**Table 5:** Estimated Extents of Heat-Induced (a) Cystine Disulfide Cleavage (Calculated via Equation 3) and (b) Cys Converted to H_2_S (Calculated via Equations 1 and 2).*.

(a) Sample	Heat treatment			
	80 °C (10 min)	90 °C (10 min)	90 °C (120 min)	UHT-like (160 °C, 160 s)
α-LA	N/A	0.10 %	0.42 %	1.44 %
Conc. α-LA	0.03 %	0.09 %	0.45 %	1.34 %
β-LG	N/A	N/A	N/A	12.15 %
Whey model	N/A	N/A	N/A	6.13 %
WPI	N/A	N/A	N/A	6.57 %

(b) Sample	Heat treatment			
	80 °C (10 min)	90 °C (10 min)	90 °C (120 min)	UHT-like (160 °C, 160 s)
β-LG	0.25%	0.93%	1.93%	1.81%
Whey model	0.37%	1.21%	2.37%	2.74%
WPI	N/A	0.52%	2.94%	3.38%
Conc. α-LA	N/A	10.74%	8.16%	29.31%

*Protein concentrations of each sample: α-LA: 2.45 mg/mL (0.17 mM); conc. α-LA: 16 mg/mL (1.11 mM); β-LG: 15.88 mg/mL (0.87 mM); whey model: 2.45 mg/mL (0.17 mM) α-LA + 15.88 mg/mL (0.87 mM) β-LG, and WPI: 30 mg/mL. N/A, not applicable.

In addition, the presence of α-LA in the mixed protein systems (i.e. the whey model and WPI) significantly facilitated LAN formation (Table 4), while a slightly higher concentration of H_2_S was also detected after heating (Table 1) as compared to the pure β-LG sample. Together with the higher H_2_S content detected in the concentrated α-LA (51.6 ± 17.9 μM) than in β-LG (22.2 ± 1.8 μM) after the UHT-like treatment, it was speculated that the total Cys content (including Cys and cystine) within the protein system promoted the formation of H_2_S.

## 4 Conclusion

H_2_S was detected in 3 % WPI solution heated at 90 °C (10 min), and the concentration of H_2_S increased significantly after more severe heat treatments (i.e. 90 °C for 120 min and the UHT-like treatment). The detected H_2_S concentrations were not only above the reported odor threshold from a previous sensory analysis (Rychlik et al., 1998), but also much higher than the previously published values detected in UHT-treated milk systems, which implied a previously neglected importance of H_2_S in the sulfur odor of heated whey solutions. However, a 10 °C decrease (to 80 °C, 10 min) significantly reduced the formation of H_2_S to below the detection limit in WPI. In the present study, protein-bound Cys residues were suggested to be the primary source of H_2_S in heated WPI, potentially formed via β-elimination, since H_2_S was detected in all β-LG-containing and Cys-containing systems, while being absent in α-LA and Met solutions with comparable concentrations to the 3 % WPI solution. The heat-induced H_2_S formation, together with its sulfurous off-flavor, is likely to have higher significance in WPI-based beverages due to a higher protein-bound Cys concentration in these products as compared to milk-based systems with a comparable protein concentration.

Disulfide cleavage, likely occurring via β-elimination of cystine residues, was also observed in intensely (≥90 °C) heated protein samples, where the increased content of free Cys residues presumably facilitated the formation of H_2_S. Under the UHT-like treatment, disulfide cleavage in β-LG-containing systems (disulfide cleavage extent: β-LG > WPI ≈ whey model system) was suggested to be more favorable compared to Cys residues being converted into H_2_S (extent of H_2_S formation: WPI > whey model system > β-LG). However, due to the reactive nature of Cys residues, β-elimination of both Cys and cystine was speculated to compete with other reactions, which potentially affected the formation of H_2_S.

## Declaration of Competing Interest

The authors declare the following financial interests/personal relationships which may be considered as potential competing interests:

Marianne Nissen Lund reports financial support was provided by Innovation Fund Denmark. Chengkang Li reports financial support was provided by China Scholarship Council. Søren Bang Nielsen reports a relationship with Arla Foods Ingredients that includes: employment. Peter Aasted Paulsen reports a relationship with Arla Foods Ingredients that includes: employment.

The remaining authors declare that they have no known competing financial interests or personal relationships that could have appeared to influence the work reported in this paper.
