# Supplementary material for: Cysteine residues are responsible for the sulfurous off-flavor formed in heated whey protein solutions
Source: Food Chem (Oxf). 2022 Jul 12;5:100120. doi: 10.1016/j.fochms.2022.100120 (PMC9294045; doi:10.1016/j.fochms.2022.100120)
Supplement: Supplementary data 1 [file mmc1.docx]

**Supplementary material**

**Cysteine residues are responsible for sulfurous off-flavor formed in heated whey protein solutions**

Chengkang Li^1^, Peter A. Paulsen^2^, Halise Gül Akıllıoğlu^1^, Søren B. Nielsen^2^, Kasper Engholm-Keller^1^, Marianne N. Lund^1, 3*^

^1^Department of Food Science, Faculty of Science, University of Copenhagen, Rolighedsvej 26, 1958 Frederiksberg, Denmark

^2^Arla Foods Ingredients, Sønderupvej 26, 6920 Videbæk, Denmark

^3^Department of Biomedical Sciences, Faculty of Health and Medical Sciences, University of Copenhagen, Blegdamsvej 3, 2200 Copenhagen N, Denmark

^*^Corresponding author: Marianne N. Lund, tel.: +4535333547, e-mail address: [mnl@food.ku.dk](mailto:mnl@food.ku.dk)

**Contents**

**Figure S1** Oxidative Amide Bond Lysis at Protein-Bound Dehydroalanine (DHA)

**Figure S2** Relationship between the Concentrations of H_2_S and LAN in Heated Whey Protein Solutions

**Table S1** LC-MS/MS Relative Quantification of LAN and LAL Cross-Linked Peptides in Heat-Treated Protein Samples

**Figure S1:** Oxidative amide bond lysis at protein-bound DHA. Adapted from (Cohen et al., 2007; Herbert et al., 2003). The dashed line indicates the cleavage point.


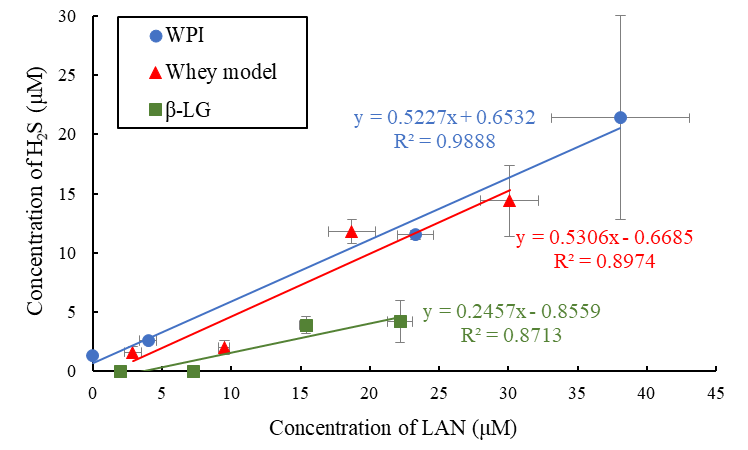


**Figure S2:** Relationship between concentrations of H_2_S and LAN in heated whey protein solutions (WPI [blue circles]; the whey model [red triangles]; and β-LG [green squares]). The values and the corresponding standard deviations can be found in **Tables 1** and **4**. There are four data points for each samples referring to different heating conditions (80 °C, 10 min; 90 °C, 10 min; 90 °C, 120 min; and 160 °C, 160 s, respectively).

**Table S1 LC-MS/MS Relative Quantification of LAN and LAL Cross-Linked Peptides in Heat-Treated Protein Samples.***

| **Modification** | **Protein(s)** | **Cross-linked residues** | **Sample** | **Heat Treatment** | | | | |
| --- | --- | --- | --- | --- | --- | --- | --- | --- |
|  |  |  |  | **Unheated** | **80 °C, 10 min** | **90 °C, 10 min** | **90 °C, 120 min** | **UHT-like (160 °C, 160 s)** |
| HAL | α-LA - α-LA | Cys91-His107 (mis-cleaved) | α-LA | ND | ND | ND | 21.6 ± 2.5% ^a^ | 100.0 ± 16.3% ^b^ |
|  |  |  | whey model | ND | ND | ND | ND | ND |
|  |  |  | WPI | ND | ND | ND | ND | ND |
| LAL | α-LA - α-LA | Cys91-Lys98 (mis-cleaved) | α-LA | ND | ND | ND | 3.2 ± 0.8% ^a^ | 100.0 ± 7.5% ^b^ |
|  |  |  | whey model | ND | ND | ND | ND | ND |
|  |  |  | WPI | ND | ND | ND | ND | ND |
| LAN | α-LA - α-LA | Cys61-Cys111 (mis-cleaved) | α-LA | ND | ND | 3.1 ± 1.0% ^a^ | 52.7 ± 7.9% ^b^ | 47.0 ± 7.8% ^b^ |
|  |  |  | whey model | ND | ND | ND | 100.0 ± 19.7% ^d^ | ND |
|  |  |  | WPI | ND | ND | ND | 72.6 ± 13.3% ^c^ | ND |
|  |  | Cys111-Cys120 (mis-cleaved) | α-LA | ND | ND | 1.5 ± 0.6% ^a^ | 25.1 ± 1.8% ^c^ | 100.0 ± 7.0% ^e^ |
|  |  |  | whey model | ND | ND | ND | 40.8 ± 7.0% ^d^ | ND |
|  |  |  | WPI | ND | ND | ND | 17.6 ± 2.2% ^b^ | ND |
|  |  | Cys6-Cys120 (mis-cleaved) | α-LA | 87.6 ± 4.6% ^b^ | 74.3 ± 3.1% ^a^ | 75.6 ± 2.1% ^a^ | 100.0 ± 3.8% ^c^ | 86.9 ± 4.5% ^b^ |
|  |  |  | whey model | ND | ND | ND | ND | ND |
|  |  |  | WPI | ND | ND | ND | ND | ND |
|  |  | Cys68-Cys28 (mis-cleaved) | α-LA | 7.1 ± 0.6% ^a^ | 7.8 ± 0.8% ^a^ | 11.9 ± 2.9% ^a^ | 72.3 ± 5.7% ^b^ | 100.0 ± 10.0% ^c^ |
|  |  |  | whey model | ND | ND | ND | 69.5 ± 6.9% ^b^ | ND |
|  |  |  | WPI | ND | ND | ND | 8.1 ± 3.2% ^a^ | ND |
|  |  | Cys6-Cys61 (mis-cleaved) | α-LA | ND | ND | ND | 100.0 ± 6.9% ^b^ | 54.1 ± 9.5% ^a^ |
|  |  |  | whey model | ND | ND | ND | ND | ND |
|  |  |  | WPI | ND | ND | ND | ND | ND |
|  |  | Cys73/Cys77-Cys111 (mis-cleaved) | α-LA | ND | ND | ND | 34.3 ± 3.3% ^b^ | 100.0 ± 8.4% ^d^ |
|  |  |  | whey model | ND | ND | ND | 86.0 ± 13.3% ^c^ | ND |
|  |  |  | WPI | ND | ND | ND | 21.6 ± 16.8% ^a^ | ND |
|  |  | Cys6-Cys73/Cys77 (mis-cleaved) | α-LA | ND | ND | ND | ND | 100.0 ± 13.3% |
|  |  |  | whey model | ND | ND | ND | ND | ND |
|  |  |  | WPI | ND | ND | ND | ND | ND |
|  |  | Cys61-Cys73/Cys77 | α-LA | 0.2 ± 0.0% ^a^ | 0.9 ± 0.2% ^a^ | 3.4 ± 1.5% ^a^ | 39.8 ± 1.9% ^d^ | 100.0 ± 4.0% ^e^ |
|  |  |  | whey model | ND | ND | ND | 30.8 ± 5.9% ^c^ | ND |
|  |  |  | WPI | ND | ND | ND | 17.4 ± 5.2% ^b^ | ND |
|  |  | Cys91-Cys111 (mis-cleaved) | α-LA | ND | ND | 0.9 ± 0.3% ^a^ | 17.1 ± 1.9% ^b^ | 100.0 ± 15.5% ^d^ |
|  |  |  | whey model | ND | ND | ND | 43.4 ± 5.8% ^c^ | ND |
|  |  |  | WPI | ND | ND | ND | 20.5 ± 4.9% ^b^ | ND |
|  | β-LG - β-LG | Cys66-Cys106 | β-LG | ND | 4.7 ± 0.8% ^a^ | 6.0 ± 1.6% ^a^ | 45.4 ± 6.6% ^b^ | 100.0 ± 19.0% ^d^ |
|  |  |  | whey model | ND | 10.3 ± 2.2% ^a^ | 10.7 ± 1.4% ^a^ | 37.9 ± 5.2% ^b^ | 65.8 ± 15.8% ^c^ |
|  |  |  | WPI | 39.7 ± 6.5% ^b^ | 41.7 ± 9.9% ^b^ | 49.8 ± 7.3% ^b,c^ | 96.6 ± 14.9% ^d^ | 84.4 ± 22.1% ^d^ |

*The results of LAN and LAL are presented as percentage values (the normalized peak area of the cross-linked peptide for a particular treatment divided by the highest normalized peak area of the same cross-linked peptide among all treatments and samples) with their respective standard deviations (SDs) in different heat-treated protein samples from the reduced sample group. Values from the same cross-link followed by different letters are significantly different between treatments and systems (one-way ANOVA with a Tukey-Kramer HSD post-hoc test, p<0.05, n=3). ND, not detected.

# References

Cohen, S. L., Price, C., & Vlasak, J. (2007). β-Elimination and Peptide Bond Hydrolysis: Two Distinct Mechanisms of Human IgG1 Hinge Fragmentation upon Storage. *Journal of the American Chemical Society*, *129*(22), 6976–6977. https://doi.org/10.1021/ja0705994

Herbert, B., Hopwood, F., Oxley, D., McCarthy, J., Laver, M., Grinyer, J., Goodall, A., Williams, K., Castagna, A., & Righetti, P. G. (2003). β-elimination: An unexpected artefact in proteome analysis. *Proteomics*, *3*(6), 826–831.

https://doi.org/10.1002/pmic.200300414
